# Telemedizin bei Fazialisparese: Aktuelle Entwicklungen und Möglichkeiten bei der Behandlung in der HNO-Heilkunde

**DOI:** 10.1007/s00106-024-01449-4

**Published:** 2024-03-26

**Authors:** Jonas Ballmaier, Sabrina Hölzer, Maren Geitner, Anna-Maria Kuttenreich, Christian Erfurth, Orlando Guntinas-Lichius, Gerd Fabian Volk

**Affiliations:** 1 Klinik und Poliklinik für Hals‑, Nasen- und Ohrenheilkunde, Institut für Phoniatrie und Pädaudiologie, Universitätsklinikum Jena, Jena, Deutschland; 2 Fazialis-Nerv-Zentrums Jena, Klinik und Poliklinik für Hals‑, Nasen- und Ohrenheilkunde, Institut für Phoniatrie und Pädaudiologie, Universitätsklinikum Jena, Haus A, Am Klinikum 1, 07747 Jena, Deutschland; 3 Zentrum für Seltene Erkrankungen Jena, Universitätsklinik Jena, Jena, Deutschland; 4 Fachbereich Wirtschaftsingenieurswesen, Ernst-Abbe-Hochschule Jena, Jena, Deutschland

**Keywords:** Digitale medizinische Technologie, Bell-Lähmung, Telesprechstunde, Videokonferenz, eHealth, Digital health technology, Bell palsy, Distance counseling, Videoconferencing, eHealth

## Abstract

Auch in der Medizin gewinnt die Digitalisierung immer schneller an Bedeutung. Die COVID-19-Pandemie beschleunigte diesen Prozess zusätzlich, und die Politik versucht, Rahmenbedingungen für einen erfolgreichen Wissenstransfer und eine bessere digitale medizinische Versorgung zu schaffen. Im vorliegenden Artikel wird die Rolle der Telemedizin bei der Behandlung von Patientinnen und Patienten mit Fazialisparese erörtert. Eine Fazialisparese hat vielfältige Auswirkungen, von Einschränkungen der Gesichtsbeweglichkeit bis zu psychologischen Folgeerkrankungen. Während viele der akuten idiopathischen Fazialisparesen sich nach einigen Wochen bessern, entwickeln etwa ein Drittel der Betroffenen Synkinesien, unwillkürliche Mitbewegungen, welche lebenslange funktionelle und psychologische Folgen haben. Die Therapie umfasst verschiedene Modalitäten, über Medikamente und Chirurgie bis zum Bewegungstraining. Bei regionaler Unterversorgung, aber auch in der Versorgung chronischer Fazialisparesen, bietet die Telemedizin innovative Lösungsansätze. Der Artikel definiert den Begriff „Telemedizin“ im aktuellen Kontext und zeigt verschiedene Anwendungsarten auf. Eine detaillierte Analyse der Anwendungsszenarien von Telemedizin bei Patientinnen und Patienten mit Fazialisparese zeigt, dass trotz geringer Evidenz viele potenziell nützliche Konzepte existieren.

## Abstract (en)

Digitalization is also becoming increasingly important in medicine. The COVID-19 pandemic has further accelerated this process and politicians are trying to create a framework for successful knowledge transfer and better digital medical care. This article describes the role of telemedicine in the treatment of patients suffering from facial nerve palsy. Facial nerve palsy has a wide range of effects, from limitations in facial mobility to psychological sequelae. While many of the acute, idiopathic facial nerve palsies improve after a few weeks, around a third of those affected develop synkinesis, involuntary movements that have lifelong functional and psychological consequences. Treatment includes various modalities, from medication and surgery to movement training. Telemedicine offers innovative solutions in cases of regional underuse, but also in the treatment of chronic facial nerve palsies. The article defines the term “telemedicine” in the current context and presents different types of application. A detailed analysis of the application scenarios of telemedicine in facial nerve palsy patients shows that despite a lack of evidence, many potentially useful concepts exist.

## Telemedizinische Behandlung

In der Medizin gewinnt die Digitalisierung immer schneller an Bedeutung. Damit wird auch in der HNO-Heilkunde die Telemedizin immer wichtiger [3]. Dieser Prozess wurde durch COVID-19 deutlich beschleunigt [13]. Die Digitalisierungsstrategie des Bundes für das Gesundheitswesen versucht diesen Prozess zu regeln und den Transfer von Wissen und medizinischer Versorgung voranzutreiben [6].

Die Rolle von telemedizinischen Methoden in der HNO-Heilkunde wurde bereits in mehreren Übersichtsartikeln dargelegt [3, 13, 16]. Hierzu ergänzend werden nun Optionen der telemedizinischen Behandlung von Patientinnen und Patienten mit Fazialisparese dargestellt sowie aktualisierte regulatorische und technische Aspekte erläutert.

## Fazialisparese

Die Folgen einer Gesichtsnervenlähmung sind vielfältig und z. T. erheblich: Einschränkungen der Gesichtsbeweglichkeit mit fehlendem Augenschluss können zu Folgeschäden der Kornea führen; eine fehlerhafte Kontrolle der Lippen erschwert Sprechen und Nahrungsaufnahme. Psychologische Folgen durch die oft deutlich sichtbare Stigmatisierung führen zu sozialen Vermeidungsstrategien [21].

### Formen

Die häufigste Form ist die idiopathische Fazialisparese – auch Bell-Parese oder Bell-Lähmung – die im Grad der Lähmung von leichter Schwäche bis hin zu vollständiger Paralyse reicht. Typischerweise setzt diese schnell, z. B. über Nacht, ein und erreicht innerhalb weniger Tage ihren höchsten Schweregrad. Die Rückbildung erfolgt langsam und kann zwischen einigen Wochen und bis zu einem halben Jahr dauern [17, 31].

Eine Ursache der Fazialisparese lässt sich in etwa 30 % der Fälle identifizieren, hierzu gehören u. a. Infektionen, Traumata, Neoplasien und systemische Erkrankungen [17].

### Phasen

Die meisten Betroffenen erlangen ihre normale Gesichtsbeweglichkeit wieder. Jedoch entwickelt knapp ein Drittel Synkinesien, bei denen es zu einer unwillkürlichen Mitbewegung ipsilateraler Gesichtsmuskeln kommt. Diese beruhen auf einer fehlerhaften Reinnervation der mimischen Muskulatur durch fehlgeleitete Axone des N. facialis [12].

Ausgeprägte Synkinesien haben erhebliche funktionelle, soziale und psychologische Folgen

Ausgeprägte Synkinesien haben erhebliche funktionelle, soziale und psychologische Folgen, die denen einer akuten Fazialisparese nicht unähnlich sind [21]. So können durch das Gegenspiel synchron fehlaktivierter Muskelgruppen Essen, Sprechen und Lächeln erschwert sein, durch Hyperfunktion des M. orbicularis oculi Sehstörungen entstehen und durch die einseitige Muskelhypertonie Gesichtsasymmetrien und Verspannungen folgen [12]. Entsprechend dem Pathomechanismus entwickeln sich Synkinesien erst 3–6 Monate nach Beginn der schlaffen Lähmung und bleiben danach ein Leben lang bestehen [19].

Bei der peripheren Fazialisparese nimmt die phasengerechte Therapie eine zentrale Rolle ein [25]. Bei der akuten idiopathischen Parese steht eine zeitnahe medikamentöse Therapie mit Steroiden im Mittelpunkt [15]. In anderen Fällen sollte die zugrunde liegende Pathologie mitbehandelt werden. In der paralytischen oder schlaffen Phase sollten außerdem eine Patientenedukation mit Instruktionen zu Augenschutz sowie Kompensationsstrategien erfolgen. Bewegungstraining, Aufklärung über normale Bewegungsmuster, Reedukation von sachten, symmetrischen und isolierten Bewegungen mit Spiegeltraining oder anderen Biofeedbackmechanismen helfen in der paretischen Phase. Sollten sich im Rahmen einer Defektheilung bei einer chronischen Parese Synkinesien entwickeln, so ist die Aufklärung der Patientin und des Patienten über die Pathomechanismen der Synkinesien und autoparalytischen Phänomenen Grundlage der Therapie. Diese umfasst außerdem u. a. die Reedukation des normalen Ruhetonus und von gezielten Gesichtsbewegungen ohne unwillkürliche Mitbewegungen, manuelle Techniken wie Dehnung hypertoner Muskulatur, Biofeedbacktechniken und ggf. Chemodenervation [28, 32].

### Versorgungsdefizit

Insbesondere im Bereich chronischer Fazialisparesen mit Defektheilungen gibt es in Deutschland ein Versorgungsdefizit. Durch einen Mangel an spezialisierten Gesichtstherapeuten und nur wenige auf Fazialisparesen spezialisierte Zentren müssen Betroffene oft lange Fahrtwege in Kauf nehmen [26].

Regionale Unterversorgungen zeigen die Bedeutung der Digitalisierung im Gesundheitswesen besonders

Im Fall regionaler Unterversorgungen wird die Bedeutung der Digitalisierung im Gesundheitswesen besonders deutlich. Lange Wartezeiten, mögliche Fehldiagnosen und Folgekosten durch Diagnose- und Therapieverzögerung sind im Fall der chronischen Fazialisparese jedoch noch nicht ausreichend untersucht. In diesem Kontext steht ein Mangel an Versorgungsangeboten in abgelegenen Regionen und die Notwendigkeit einer personalisierten Therapie im Vordergrund. Telemedizinische Angebote können dazu beitragen, diese Versorgungslücken zu schließen und die Lebensqualität von Menschen mit einer chronischen Fazialisparese erheblich zu verbessern.

## Begriffsklärung „Telemedizin“

Als Nächstes erfolgt die Einordnung des Begriffs „Telemedizin“ im Kontext von „Digital Health“. Obwohl es bereits zahlreiche Definitionen gibt, bleibt es von entscheidender Bedeutung, die begriffliche Verortung von Telemedizin zu präzisieren und die vielfältigen Unterscheidungen in diesem Bereich zu beleuchten. Diese Notwendigkeit resultiert insbesondere aus den kontinuierlichen Veränderungen und den anhaltenden Begriffsunsicherheiten im Bereich der digitalen Gesundheit. In diesem Abschnitt ist es Ziel der Autorinnen und Autoren, den Begriff Telemedizin einzuordnen, zu definieren und die gängigsten Klassifikationen und Unterscheidungen darzustellen. Dies schafft die Grundlage für ein gemeinsames Verständnis und ermöglicht die Erfassung des aktuellen Stands in Forschung und Praxis.

Digitale Gesundheit (Digital Health) hat sich zu einem maßgeblichen Bereich entwickelt, die verschiedenen Facetten der modernen Gesundheitsversorgung umfasst und sich auf die Anwendung von innovativen Technologien zur Verbesserung von Gesundheit und Gesundheitsmanagement bezieht. Der Begriff „digitale Gesundheit“ wird heutzutage zunehmend als umfassender Oberbegriff verwendet. Er schließt nicht nur eHealth (elektronische Gesundheit) und mHealth (mobile Gesundheit) ein, sondern erstreckt sich auch auf aufstrebende Felder wie den Einsatz fortschrittlicher Informatikwissenschaften. Dies umfasst beispielsweise das Internet der Dinge, Big Data, Genomik, Robotik und künstliche Intelligenz (KI) [36]. Ziele sind u. a. die fortschreitende Digitalisierung von Gesundheitsdaten, der effektive und sichere Einsatz von Telemedizin und die Integration von Technologien zur Unterstützung von Gesundheitsdienstleistungen.

Die Weltgesundheitsorganisation (WHO) betont dabei, dass eHealth („electronic health“) die kosteneffiziente und sichere Nutzung von Informations- und Kommunikationstechnologien (IKT) zur Unterstützung von Gesundheit und gesundheitsbezogenen Bereichen umfasst. Diese Bereiche schließen Gesundheitsdienste, Gesundheitsüberwachung, Gesundheitsliteratur sowie Gesundheitsbildung, Wissensvermittlung und Forschung ein. Das Ziel besteht darin, dass die Stärkung von Gesundheitssystemen durch eHealth dazu beitragen kann, die Wahrnehmung grundlegender Menschenrechte zu fördern, indem sie die Gerechtigkeit, Solidarität, Lebensqualität und Qualität der Versorgung verbessert [34].

mHealth ist eine Schlüsselkomponente von eHealth, die sich auf die Nutzung mobiler drahtloser Technologien für die öffentliche Gesundheit konzentriert. Hierbei werden mobile Geräte und drahtlose Kommunikation genutzt, um den Zugang zu Gesundheitsinformationen und -dienstleistungen zu verbessern. mHealth spielt eine entscheidende Rolle in der Förderung von Gesundheitspraktiken und der Erweiterung des Gesundheitsmanagements über mobile Plattformen [35].

Eine weit verbreitete Kategorie von eHealth ist „eCare“, die in der Gesundheitsversorgung verschiedene Bereiche umfasst. Telemedizin kann im Rahmen von eHealth unter eCare subsumiert werden. Nach der Definition der Bundesärztekammer ist Telemedizin „ein Sammelbegriff für verschiedenartige ärztliche Versorgungskonzepte“ und umfasst verschiedene ärztliche Leistungen in den Bereichen Diagnostik, Therapie, Rehabilitation und ärztliche Entscheidungsberatung, die über räumliche Distanzen hinweg erbracht werden [5].

Telemedizinische Methoden sind integraler Bestandteil nahezu jeden medizinischen Fachgebiets

Marx et al. analysieren in ihrem Beitrag den Unterschied zwischen 2 Definitionen von Telemedizin, wobei sie insbesondere darauf hinweisen, dass die WHO den Begriff „Telemedizin“ auf alle Berufsgruppen anwendet, während die Bundesärztekammer (BÄK) sich vornehmlich auf ärztliche Leistungen konzentriert (Tab. 1; [23]). Beide Ausführungen unterstreichen die Bedeutung, dass die Einführung eines eigenen Begriffs eher auf die Existenz eines eigenständigen Forschungs- oder Fachbereichs hinweist. Laut BÄK sollte dieser Ansatz jedoch vermieden werden, da telemedizinische Methoden integraler Bestandteil nahezu jeden medizinischen Fachgebiets sind, was im Sinne einer umfassenden Gesundheitsversorgung der Bevölkerung betont wird. Zusammenfassend kann festgestellt werden, dass Telemedizin oder telemedizinische Anwendungen einen klaren Schwerpunkt auf die direkte Patientenversorgung legen und digitale Technologien nutzen, um medizinische Informationen über Entfernungen hinweg auszutauschen. Diese sollten von infrastrukturellen Themen im Bereich der Telematik deutlich abgegrenzt werden. Der Begriff Telematik verbindet Telekommunikation und Informatik. Er bildet die essenzielle Grundlage für die Telematikinfrastruktur (TI). Die TI ist ein umfassendes System, das der sicheren Vernetzung verschiedener Akteure im deutschen Gesundheitswesen dient [10].

**Tab. 1:** Bereiche und Vergleich der Definitionen zur Telemedizin durch Bundesärztekammer (BÄK) und Weltgesundheitsorganisation (WHO). (In Anlehnung an [5, 10, 20, 23, 34])

eHealth		Beispiele	BÄK	WHO
*eCare*	Gesundheitsversorgung	Telekonsil, Telekonsultation (z. B. Videosprechstunde), Telemonitoring/Remote-Patient-Management	Ja	Ja
eAdministration*	Administrative Prozesse	Elektronische Gesundheitskarte (eGK), elektronischer Heilberufsausweis (HBA), elektronische Patientenakten (ePA), eRezept, elektronische Arbeitsunfähigkeitsbescheinigung (eAU), elektronischer Medikationsplan (eMP), Kommunikation im Medizinwesen (KIM), Notfalldatenmanagement (NFDM)	Nein*	Nein*
*ePrevention*	Prävention	Altersgerechte Assistenzsysteme (AAL), Coaching	Nein	Ja
*eResearch*	Forschung	Genomforschung mittels IKT usw.	Nein	Ja
*eLearning*	Lehre	Blended Learning (z. B. ILIAS)	Nein	Ja

*Ermöglicht durch Telematikinfrastruktur (TI)

*IKT *Informations- und Kommunikationstechnologien, *ILIAS* E-Learning-Plattform

## Anwendungsbereiche der Telemedizin

Die verschiedenen Anwendungsarten der Telemedizin können in Deutschland in verschiedene Kategorien unterteilt werden [5, 20, 23].

### Kategorien

*Telekooperation/-konsil* bezeichnet die simultane oder zeitversetzte Kommunikation zwischen anfragenden und konsultierenden Fachkräften im Gesundheitswesen. Dieser Austausch erfolgt auf elektronischem Wege und betrifft eine patientenbezogene medizinische Fragestellung. Alternativ kann ein Videokonsil durchgeführt werden, an dem auch die Person mit medizinischem Bedarf teilnimmt.

*Telediagnostik* (wie Teleradiologie oder Telepathologie): In der bildgestützten Telediagnostik werden medizinische Bilder von räumlich voneinander entfernten Teilnehmern zur Diagnosestellung befundet.

*Teletherapie/-konsultation* (z. B. via Videosprechstunde) bezeichnet die ärztliche oder therapeutische Behandlung des Patienten über Telekommunikation. Auch zwischen Patientinnen und Patienten und Therapeutinnen und Therapeuten kann es eine sinnvolle Ergänzung zur herkömmlichen physischen Therapie in der Praxis darstellen. Die von der Bundesärztekammer in dem Zusammenhang verwendete Bezeichnung als Telekonsultation lässt nur eine schwere semantische Differenzierung zu Telekonsilen zu. Auch die Bezeichnung als Teletherapie erlaubt nur eine kontextbezogene Differenzierung zur homonymen Begrifflichkeit in der Strahlentherapie [4].

*Telemonitoring* (z. B. telekardiologische Anwendungen via Apps, z. B. bei Herzrhythmusstörungen, Bluthochdruck, Asthma, Diabetes) bezeichnet die ferngesteuerte Überwachung und Analyse von Gesundheitsparametern, wobei diese elektronisch übertragen werden. Telemonitoring zeigt das Potenzial, die Behandlung von chronisch kranken Menschen zu optimieren, und kann sowohl präventiv als auch als Begleitung einer Therapie eingesetzt werden. Diese Überwachung erfolgt häufig im häuslichen Umfeld der Patientinnen und Patienten und nicht in einer medizinischen Einrichtung.

*Tele-Hausbesuch/-Visite *bezeichnet die Vor-Ort-Betreuung durch qualifiziertes medizinisches Personal (nichtärztliche Praxisassistenz – NäPa), während die Ärztin oder der Arzt per Videokonferenz zugeschaltet werden kann. Dadurch erhalten Patientinnen und Patienten Zugang zu ärztlicher Versorgung, der ihnen ansonsten nur schwer zugänglich wäre. Dieses Versorgungskonzept wird derzeit in ausgewählten Regionen Deutschlands getestet [1].

### Zeitliche Dimension der Kommunikation

Diese Kategorien repräsentieren verschiedene Aspekte und Anwendungen der Telemedizin im Gesundheitswesen. In diesem Zusammenhang ist es von Bedeutung, die zeitliche Dimension zu berücksichtigen, in der die Kommunikation stattfindet.

Bei zeitgleicher Interaktion handelt es sich um eine *synchron* verlaufende Kommunikation

Bei zeitgleicher Interaktion, wie beispielsweise in einer Online-Videosprechstunde, handelt es sich um eine *synchron* verlaufende Kommunikation. Hingegen wird bei einer Datenspeicherung und zeitlich verzögerten Auswertung oder Übermittlung, wie sie im Telemonitoring auftreten kann, von *asynchroner* Kommunikation gesprochen.

**Abb. 1:**
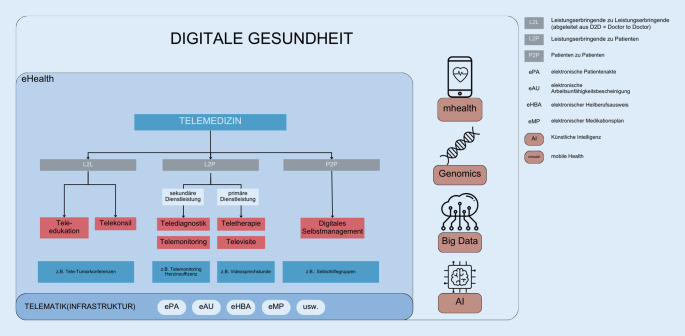
Verortung und Anwendungsbereiche der Telemedizin. (Eigene Darstellung in Anlehnung an [14, 22, 27, 35])

Die Abb. 1 illustriert die genannten Begrifflichkeiten, ordnet sie räumlich ein und setzt sie in einen zusammenhängenden Kontext. Dabei erfolgt eine Unterscheidung hinsichtlich der Beteiligten bei der Leistungserbringung, beispielsweise zwischen den Leistungserbringenden und der Patientin oder dem Patienten, wie es bei der Teletherapie der Fall ist.

### Vor- und Nachteile

Vorteile und Nachteile telemedizinischer Konzepte, welche durch den Einsatz aus Sicht der verschiedenen Beteiligten erreicht werden können, sind in Tab. 2 aufgeführt.

**Tab. 2:** Vorteile und Nachteile von telemedizinischen Angeboten. (Eigene Darstellung in Anlehnung an [13])

	Vorteile	Nachteile
*Patienten*	Erhöhte Zugänglichkeit zur Versorgung und zu Informationen unabhängig vom regionalen Angebot, schnellere Beratung und frühere Intervention, Zeit- und Kostenersparnis kontinuierlichere Betreuung, besonders bei chronischen Erkrankungen oder Rehabilitation, Diskretion und Komfort, höhere Compliance bzw. Therapieadhärenz, wenn das Angebot technisch und therapeutisch sehr gut gemacht ist	Mangel an persönlichem Kontakt, auch der Austausch mit anderen Betroffenen fehlt, begrenzte physische Untersuchung, technologische Hürden und damit drohende Ausgrenzung von technologiefernen Bevölkerungsschichten („information communication technology [ICT] illiterates“)
*Leistungserbringer*	Erweiterte Patientenversorgung und Effizienzsteigerung, Zeitersparnisse bei Vorbesprechung und Befundbesprechung, Konsultation und Zweitmeinungen bei komplexen Fällen, Telediagnostik zur Beurteilung des Fortschritts und der Wirksamkeit, effizientere Gestaltung von Genesungsprozessen und der Überwachung der Rehabilitation, Wissenstransfer und flexiblere Arbeitszeiten	Begrenzte Diagnose- und Interventionsmöglichkeiten, Haftungsfragen in Bezug auf Fernbetreuung und mögliche Diagnose- und Behandlungsfehler, Kosten- und Zeitaufwand für die Implementierung, Schulungen und Aufrechterhaltung, Datenschutz und Sicherheitsfragen
*Kostenträger*	Effiziente Versorgungskoordination und Kostenreduktion, bessere Patientenergebnisse und erhöhte Zufriedenheit, Reduktion von Ansteckungsgefahr, besseres Gesundheitsmanagement und schnelle Adaption digitaler Gesundheitsangebote	Notwendigkeit von Qualitätskontrollen, Herausforderungen bei der Integration und Implementation

## Anwendungsszenarien bei Fazialisparese

Für den Anwendungsfall von Telemedizin bei der Fazialisparese finden sich nur wenige Studien, und es besteht erheblicher Forschungsbedarf. De Jongh et al. fassten in einem Review 6 Studien zum Einsatz der Telemedizin in Bezug auf die Behandlung von Fazialisparesen zusammen. Dort werden telemedizinische Konzepte unterteilt in eHealth, im Sinne von Videosprechstunden, und mHealth, als durch mobile Geräte unterstützte medizinische Anwendungen. Die meisten elektronischen und mobilen Gesundheitsanwendungen konzentrieren sich auf die Bildgebung, werden aber in der täglichen klinischen Praxis nicht in großem Umfang eingesetzt [7].

Synchrone Anwendungen der Telemedizin bei Fazialisparese werden u. a. im Rahmen ärztlicher Videosprechstunden beschrieben. In einer retrospektiven Analyse von Erstvorstellungen im Rahmen einer Videosprechstunde eines spezialisierten Zentrums konnte bei den meisten Patientinnen und Patienten bereits eine Diagnose gestellt und Therapien empfohlen werden [26]. Die Möglichkeit einer Fernbehandlung durch Gesichtstherapeutinnen und -therapeuten wurde in einem Case-Review gezeigt, in dem einer ambulanten Behandlung ein telemedizinisch begleitetes Training nachgestellt wurde [8]. In einer Analyse von „patient-reported outcome measures“ nach 10 telemedizinischen Therapieeinheiten bei Patientinnen und Patienten mit orookulären Synkinesien zeigte sich eine Verbesserung im Facial Disability Index [18].

Eine asynchrone Evaluation der Gesichtsbeweglichkeit ist anhand von Videoaufzeichnungen möglich

Eine asynchrone Evaluation der Gesichtsbeweglichkeit nach Fazialisparese ist anhand von Videoaufzeichnungen möglich. In einer explorativen Studie zeigt sich eine ähnliche Zuverlässigkeit in der Einschätzung der Schweregrade nach House-Brackmann- und Sunnybrook-Skala in Videoaufzeichnungen im Vergleich zur persönlichen Bewertung, jedoch mit einer unzureichenden und nicht zuverlässigen Übereinstimmung zwischen den Methoden, insbesondere bei der Beurteilung von Synkinesien. Hierbei wurde auch betont, dass die asynchrone Bewertung den Einfluss von Echtzeitinteraktionen während telemedizinischer Bewertungssitzungen nicht berücksichtigt [30].

mHealth-Anwendungen bieten synchrone und asynchrone Konzepte. Erfahrungen mit einem App-Prototyp bei der Beurteilung gesunder Teilnehmer unter Verwendung einer TrueDepth-Kamera (Smartphone-Kamera, welche Tiefeninformationen liefert) zeigen ein erhebliches Potenzial für die App-gestützte, automatisierte Einstufung von Gesichtsbewegungsstörungen. Prospektive klinische Studien zur Evaluation und zur Korrelation mit gängigen Gesichtsscores stehen aus [29].

Noch weniger automatisiert ist die IOS-App „eFACE“ („electronic, clinician-graded facial function scale“), bei der auf einem iPhone oder iPad die Schwere einer Fazialisparese auf 16 Subskalen in Prozentschritten von den Bewertenden eingegeben werden. Die App errechnet Summenwerte und dokumentiert diese. Eine Weiterentwicklung, die mittels einer Kamera und einer geschulten KI automatisch die Schwere erkennt (Arbeitstitel: „Auto-eFACE“), ist in Arbeit [2].

Tragbare Elektromyographie(EMG)-Systeme für den Heimgebrauch mit Cloud-Anbindung zur Überwachung von Übungen und zur Dokumentation des Fortschritts im Sinne eines Telemonitorings befinden sich zumindest in der Konzeptionierung [33].

Insgesamt zeigen sich für den Einsatz telemedizinischer Konzepte in Bezug auf die Fazialisparese eine geringe Evidenz. Es existieren keine prospektiven klinischen Studien. Die meisten Anwendungen sind bislang in der Entwicklung nicht über ein Proof-of-Concept-Status hinaus evaluiert. Ob und wie sich die Versorgungsqualität durch die Telemedizin bei Betroffenen beeinflussen lässt, sollte anhand qualitativ hochwertigerer Untersuchungen gezeigt werden.

An welcher Stelle Patientinnen und Patienten mit einer Fazialisparese schon jetzt während ihrer Krankheit mit Telemedizin in Kontakt kommen könnten, zeigt eine fiktive „patient journey“ in Tab. 3.

**Tab. 3:** Fiktive „patient journey“.Zur Erläuterung der heutigen möglichen Rolle von Telemedizin für Patientinnen und Patienten mit Fazialisparese

Eine 42-jährige Patientin bemerkt beim abendlichen Blick in den Spiegel eine Minderbeweglichkeit der linken Gesichtshälfte. Sie begibt sich aus Angst vor einem Schlaganfall in die nächstgelegene Notaufnahme. Es erfolgt dort eine cCT, welche *teleradiologisch befundet wird*	Teleradiologie: synchron oder asynchron möglich, Telediagnostik, Arzt-zu-Arzt und Arzt-zu-MTRA
Die Klinik verfügt über keine eigene Neurologie, ist aber als Teil eines neurovaskulären Netzwerks eine *telemedizinisch vernetzte Stroke Unit.* Nach dem Ausschluss eines Schlaganfalls erhielt die Patientin die Diagnose einer akuten peripheren Fazialisparese, eine Prednisolontherapie wurde eingeleitet. Außerdem erhält sie ein Rezept für die ambulante Logopädie zur spezialisierten Gesichtstherapie	Telekonsil: synchron oder asynchron, Arzt (Notaufnahme) zu Arzt (überregionales Schlaganfallzentrum)
*Über eine Internetsuche findet die Patientin Informationen über ihre Erkrankung* sowie über eine *Expertenliste* eine auf die Behandlung von akuten und chronischen Fazialisparesen mit orookulären Synkinesien spezialisierte Gesichtstherapeutin. Nach einer ersten Therapieeinheit in der eine Fahrstunde entfernten Praxis erfolgen die *weiteren Therapieeinheiten als Videosprechstunde*	Wissensdatenbanken: asynchron, Patientenedukation, Empowerment
Im Verlauf der nächsten Monate bemerkt die Patientin eine Besserung der Gesichtsbeweglichkeit, jedoch auch vermehrt ungewollte Muskelbewegungen sowie Verspannungen der zuvor gelähmten Gesichtsseite. Da sie sich schon in der Videotherapie mit einer spezialisierten Gesichtstherapeutin befindet, wird von dieser Therapeutin früh der Wechsel in die neue Krankheitsphase erkannt. Auf Empfehlung der Expertin erhält die Patientin vom Hausarzt eine Überweisung an ein spezialisiertes Zentrum. Das nächste findet sie in 400 km Entfernung	Videosprechstunde: synchron, Therapeut-zu-Patient
*Diese Klinik bietet auch Videosprechstunden an.* Im Rahmen der Erstvorstellung in der Videosprechstunde kann die Diagnose einer Defektheilung mit orookulären Synkinesien nach akuter Fazialisparese gestellt werden	Videosprechstunde: synchron, Arzt-zu-Patient
Um den Verlauf der Erkrankung zu dokumentieren, wurde die Patientin in eine Studie eingeschlossen, bei der eine automatisierte *Erkennung der fazialen Fehlbewegungen mithilfe einer Smartphone-Kamera erfolgt*	Telemonitoring: synchron oder asynchron, z. B. erlaubt KI-gestützte Bilderkennung asynchrone und synchrone Verlaufskontrolle
Bei einer erneuten Konsultation des spezialisierten Fazialisnervzentrums im Rahmen einer Videosprechstunde wird die Indikation zu einer Botulinumtoxin-Injektion gestellt. Die *Aufklärung erfolgt bereits während der Videosprechstunde,* und ein Termin zur Therapie und persönlichen Erstvorstellung wird vereinbart	Im Rahmen einer Videosprechstunde sind auch Aufklärungen möglich
Außerdem wird die Patientin auf Selbsthilfegruppen hingewiesen, zu denen sie über *Social-Media-Kanäle Kontakt aufnimmt und an einem ersten Treffen per Videokonferenz teilnimmt*	Social-Media-Kanäle: synchron und asynchron, Patienten-zu-Patienten

*cCT* kraniale Computertomographie, *KI* künstliche Intelligenz, *MTRA *medizinisch-technische Radiologie-Assistentin

**Tab. 4:** Checkliste für die Durchführung von Videosprechstunden für die Behandlung von Patienten mit Fazialisparese

✓	Die Nutzung eines zertifizierten Videodienstanbieters ist Voraussetzung für die Abrechnung der Leistungen nach EBM (einheitlicher Bewertungsmaßstab)
✓	In Fällen, in denen die Patientin oder der Patient unbekannt ist, können Identitätsprüfung und die Erfassung erforderlicher Daten fernmündlich durchführt werden. Eine pragmatische Lösung ist der Abgleich des Fotos und der Daten der Krankenkassenkarte am Anfang der Videosprechstunde
✓	Ausstattung mindestens Bildschirm, Kamera, Mikrofon, Lautsprecher, stabile Internetverbindung (Empfehlung: große Monitore, damit mimische Details gut erkannt werden)
✓	Geeignete ruhige Umgebung mit guten Lichtverhältnissen (Empfehlung speziell für die Beurteilung von Fazialisparesen: neutrales bzw. hartes Licht von links und rechts und nicht frontal, um mimische Bewegungen und Faltenbildung zu betonen)
✓	Einwilligung der Patientinnen und Patienten, Information der Patientinnen und Patienten

## Rahmenbedingungen

Trotz des zunehmenden Fokus auf die Digitalisierung fällt Deutschland im internationalen Vergleich immer weiter zurück. In einer Studie der Bertelsmann Stiftung, die den Digitalisierungsgrad von 17 ausgewählten westlichen Ländern untersuchte, platzierte Deutschland sich auf dem vorletzten Rang. Dieses Ergebnis wird in weiteren Studien [9, 11] bestätigt. Die Gründe für die verzögerte Digitalisierung in Deutschland sind vielfältig und umfassen neben komplexen Akteurskonstellationen, die teilweise auf die Selbstverwaltung zurückzuführen sind, auch Bürokratie, hohe Kosten für Technologie, Sicherheitsbedenken und regulatorische Unsicherheiten. Hinzu kommen mangelnde Verlässlichkeit der technischen Lösungen und fehlende Interoperabilität der Systeme, wie im Bericht „E-Health in Deutschland 2022“ dargelegt [24].

Aktuelle Entscheidungen, wie beispielsweise die Verabschiedung des Digital-Gesetzes (DigiG), das Regelungen für die Einführung der elektronischen Patientenakte für alle Versicherten und das E‑Rezept enthält, sowie des Gesundheitsdatennutzungsgesetzes (GDNG), das die Grundlagen für die Nutzung von Gesundheitsdaten in der Forschung schafft, wurden am 30. August 2023 vom Bundeskabinett beschlossen [6].

### Abrechnungsmöglichkeiten

Abrechnungsmöglichkeiten (Stand Dezember 2023) für telemedizinische Leistungen bestehen für den Anwendungsfall in der *HNO-Heilkunde* derzeit für Videosprechstunden und für Telekonsile für Vertrags- und Krankenhausärzte (Tab. 4). Diese können sowohl im gesetzlichen Krankenversicherungssystem (GKV) als auch im privaten Krankenversicherungssystem (PKV) abgerechnet werden. Die Abrechnung nach dem einheitlichen Bewertungsmaßstab (EBM) erfolgt in erster Linie über Grundpauschalen, die durch Zuschläge ergänzt werden. Dazu gehören beispielsweise Zuschläge für die fachärztliche Grundversorgung und die Anschubfinanzierung telemedizinischer Leistungen.

Diese Zuschläge unterliegen bestimmten Abrechnungsbegrenzungen

HNO-Fachärzte können, abhängig vom Patientenalter und der Art der Behandlung, spezifische Zuschlagsziffern wie 09220 und 09222 in ihrer Abrechnung berücksichtigen. Zuschläge zur Anschubfinanzierung, wie 01450 und 01451, wurden eingeführt, um den erhöhten technischen Aufwand auszugleichen. Bei der Authentifizierung neuer Patientinnen und Patienten in der Videosprechstunde kann der Mehraufwand über die 01444 in Betracht gezogen werden, da sich die notwendigen Basisdaten nicht automatisch über die elektronische Gesundheitskarte erfassen lassen. Es ist jedoch wichtig zu beachten, dass diese Zuschläge bestimmten Abrechnungsbegrenzungen unterliegen. Wenn Patientinnen und Patienten im selben Quartal keine persönliche Vorstellung bei der Ärztin bzw. beim Arzt haben und ausschließlich telemedizinisch behandelt werden, gelten Abschlagsregelungen. In der HNO-Heilkunde führt dies zu einer Kürzung der Bewertung um 30 %. Aktuell ist die Zahl der Behandlungsfälle, die ausschließlich über Video erfolgen, auf 30 % der gesamten Behandlungsfälle begrenzt. Seit dem 12. Oktober 2023 können über den 116117-Terminservice freie Termine für Videosprechstunden gemeldet werden. Ärztinnen und Ärzte und Psychotherapeutinnen und Psychotherapeuten erhalten extrabudgetäre Vergütung und einen zeitgestaffelten Zuschlag von 100, 80 oder 40 % zur Pauschale, wenn der Termin durch eine Terminservicestelle vermittelt wird.

Abrechnungsmöglichkeiten für die Videotherapie in den *Heilmittelberufen*: Ab dem 1. Oktober 2022 können gemäß der getroffenen Vereinbarung therapeutische Maßnahmen, mit Ausnahmen, als telemedizinische Leistungen erbracht und abgerechnet werden. Eine grundlegende Voraussetzung dafür ist, dass die Versicherten der Durchführung von Videobehandlungen zustimmen. Alternativ haben sie die Möglichkeit, diese abzulehnen oder ihre Zustimmung jederzeit zu widerrufen. Sollte eine Ablehnung oder ein Widerruf erfolgen, wurde festgelegt, dass die Behandlung nahtlos in der Praxis fortgesetzt werden kann. Speziell diese Festlegung ist sehr problematisch, weil eine konsequente Umsetzung zu erheblichen organisatorischen Problemen in den eng getakteten Praxisabläufen führt und Telemedizin durch bürokratisch Hürden unnötig ineffizient wird.

Zugelassene Therapeutinnen und Therapeuten können auf diese Weise bis zu 30 % aller Behandlungen als Videobehandlungen erbringen. Im Bereich der Stimm‑, Sprech‑, Sprach- und Schlucktherapie, aber auch der Ergotherapie bezieht sich dies auf Anlage 7 des Vertrags gemäß § 125 Absatz 1 SGB V, der die Versorgung mit Leistungen sowie deren Vergütung regelt.

### Voraussetzungen

Aspekte, die weiterhin berücksichtigt werden sollten:

- Die Nutzung eines zertifizierten Videodienstanbieters ist auch für die Heilmittelberufe Voraussetzung für die Abrechnung der Leistungen nach EBM.
- Die Patientinnen und Patienten müssen gesundheitlich in der Lage sein, an der telemedizinischen Leistung teilzunehmen.
- Außerdem müssen sie über die entsprechende Medienkompetenz verfügen.
- Unter Umständen kann eine Begleitperson unterstützen.
- Die erste Therapieeinheit eines Verordnungsfalls muss in Präsenz stattfinden, ebenso Erst- und Bedarfsdiagnostik sowie Verlaufskontrollen.
- Die Behandlung muss seitens der Therapeutinen bzw. der Therapeuten in den Praxisräumen erbracht und jederzeit auch persönlich dort fortgeführt werden können.

Rechtliche aktuelle Rahmenbedingungen telemedizinischer Behandlungen sind zu beachten und wurden schon in einigen Artikeln schon ausführlich beschrieben [3, 16].

## Fazit für die Praxis

- Die zunehmende Digitalisierung unseres Lebens erzeugt in der Medizin ein besonderes Spannungsfeld zwischen Chancen und Restriktionen.
- Durch die Digitalisierung können Effizienzsteigerungen erreicht werden und durch unterschiedliche Formen der Telemedizin die Verfügbarkeit und Qualität von medizinischen Leistungen enorm verbessert werden.
- Gleichzeitig ist die Medizin durch die oft komplizierte Finanzierung, die hohen Datenschutzansprüche und die staatlichen und berufsrechtlichen Regulationen träge bei der Umsetzung von Innovationen.
- Für die Diagnostik und Behandlung der Fazialisparese stehen verschiedene telemedizinische Konzepte, aktuell vorrangig Videosprechstunden, bereits jetzt zur Verfügung, werden aber nur wenig genutzt.
- Ob und wie sich die Versorgungsqualität durch die Telemedizin bei Betroffenen beeinflussen lässt, sollte anhand qualitativ hochwertigerer Studien untersucht werden.
- Zusammenfassend wird eine zunehmende Integration telemedizinischer Ansätze in der Praxis empfohlen.
- Dabei sollten synchrone Anwendungen wie ärztliche und therapeutische Videosprechstunden vorerst priorisiert werden, um auf deren Datenbasis asynchrone Anwendungen sinnvoll skalieren zu können.
